# Revisiting Dental Care and Mental Health: A Quality Improvement Project of Dental Care for Patients With Severe Mental Illness Living in a Community Rehabilitation Centre

**DOI:** 10.1192/bjo.2024.450

**Published:** 2024-08-01

**Authors:** Yuepeng Wang, Sukhdev Singh, Lucy Duah, Serge Salih, Matthew Allin

**Affiliations:** Camden and Islington NHS Trust, London, United Kingdom

## Abstract

**Aims:**

The Care Quality Commission report (Smiling matters: oral health care in care homes) showed that too many people living in care homes were not being supported to maintain and improve their oral health. Lime Tree Garden is a purpose-built care home for up to 24 adults with mental health needs supporting people with enduring mental health illness to develop basic life skills so they can live supported in the community.

This is a quality improvement project aiming to improve oral health and to reduce the burden of oral diseases in people with mental disorders and ensure timely access to dental treatment in Lime Tree Gardens through meeting the NICE quality standards of care.

**Methods:**

Auditing current implementing status of oral health procedures in place and comparing with NICE guidelines and quality standards: 1) Adults who move into a care home have their mouth care needs assessed on admission; 2) Adults living in care homes have their mouth care needs recorded in their personal care plan; 3) Adults living in care homes are supported to clean their teeth twice a day and to carry out daily care for their dentures. For each patient, their dental care plan is recorded and compared with NICE guideline.

**Results:**

There are challenges and space for improvement while implementing Oral health toolkit for adults in care homes at Lime Tree Gardens. A significant amount of patients (>90%) have unmet needs in terms having oral health.

**Conclusion:**

There is a need to address the dental health challenges in this vulnerable population with recurrent and enduring mental illness. It is important to integrate and highlight dental health, as an important part of physical heath into the overall medical management of patients with severe mental illness in residential rehabilitation psychiatry.

